# DIVERSITY MATTERS: INTERSECTIONAL INEQUITIES IN HEALTH AND WELL-BEING THROUGH AGING

**DOI:** 10.1093/geroni/igac059.688

**Published:** 2022-12-20

**Authors:** Rachel Koffer, Johanna Drewelies, Deborah Carr

## Abstract

Different positions within social hierarchies receive unequal access to resources, leading to health disparities in later life (Agenor, 2020). Research addressing inequities must increasingly account for the many social categorizations (e.g., race, gender, socioeconomic status) that affect individuals‘ lived experiences. This symposium examines the role of intersecting social contexts on health and well-being across the life course. Surachman and colleagues use the U.S.-based National Growth and Health Study to examine the intersectionality between early life socioeconomic context and race on women’s metabolic syndrome severity. Their findings have implications for societal factors leading to accelerated aging across young adulthood and early midlife. Koffer and colleagues use the U.S.-based Study of Women’s Health Across the Nation to demonstrate differential midlife exposure to types and number of major life events across race/ethnicity and education. They subsequently find that major life events increase risk of cardiovascular disease events, indicating the importance of studying the life experiences of diverse women across midlife. Drewelies and colleagues use the Germany-based Berlin Aging Study II to look at the associations among multidomain identity and social background factors on physical, cognitive, and psychological aging. Implications from their work demonstrate the interplay of diversity on health and well-being in older adulthood. Conjointly, findings indicate that intersectional identities play an important role in shaping key outcomes of human functioning across adulthood and aging. Dr. Deborah Carr will critically discuss the three contributions from a life course perspective and provide considerations for future research and policy promoting equity for diverse older adults.

